# Differential expression of CCR2 and CX_3_CR1 on CD16^+^ monocyte subsets is associated with asthma severity

**DOI:** 10.1186/s13223-019-0379-5

**Published:** 2019-11-04

**Authors:** Reem Al-Rashoudi, Gillian Moir, Mohamed S. Al-Hajjaj, Monther M. Al-Alwan, Heather M. Wilson, Isabel J. Crane

**Affiliations:** 1 School of Medicine, Medical Sciences and Nutrition, University of Aberdeen Institute of Medical Sciences, Foresterhill, Aberdeen, AB25 2ZD Scotland, UK; 2 Department of Clinical Laboratory Sciences, College of Applied Medical Sciences, King Saud University, Riyadh, Saudi Arabia; 3 Stem Cell and Tissue Re-Engineering Program, King Faisal Specialist Hospital and Research Centre, Riyadh, Saudi Arabia; 4 College of Medicine, Al-Faisal University, Riyadh, Saudi Arabia; 5 Present Address: Clinical Sciences Department, College of Medicine, University of Sharjah, Sharjah, UAE; 6 Respiratory Division, Department of Medicine, King Khalid University Hospital, Riyadh, Saudi Arabia

**Keywords:** Saudi Arabia, Chemokine receptor, Flow cytometry, CD14, Biomarker

## Abstract

**Background:**

Monocytes play an important role in immune and inflammatory diseases and monocyte subsets are predictors of disease in certain conditions. Expression of the chemokine receptors, CCR2 and CX_3_CR1 on monocyte subsets relates to their function and can be used in their characterization. Our objective was to determine whether CD14, CD16, CCR2 and CX_3_CR1 on monocyte subsets are potential indicators of asthma severity.

**Methods:**

Blood samples were collected from Saudi Arabian patients with asthma and normal healthy individuals. Six-color flow-cytometry phenotypic analysis was used to identify human blood monocyte subsets, based on their expression of CD14 and CD16 following CD45 gating. Expression of CCR2 and CX_3_CR1 was analysed on classical (CD14^++^CD16^−^), intermediate (CD14^++^CD16^+^) and non-classical (CD14^+^CD16^++^) subsets and correlated with disease severity.

**Results:**

We demonstrated a significant increase in percentage of total CD45-positive monocytes in the blood of patients with severe asthma, but the proportion of the individual monocyte subsets was not significantly changed when patients with mild, moderate and severe asthma were compared with healthy individuals. CD16 expression (mean fluorescence intensity, MFI) was decreased on intermediate and non-classical subsets in patients with severe asthma compared to healthy controls. CX_3_CR1 expression was also lower, with a lower percentage of cells expressing CX_3_CR1 in the non-classical CD14^+^CD16^++^ subset in all patients with asthma and this was inversely related to the percentage of cells expressing CCR2.

**Conclusions:**

CCR2 expression on monocytes indicated a tendency toward more phagocytic monocytes in patients with asthma. The differential expression of CD16, CX_3_CR1 and CCR2 on monocyte subsets in peripheral blood indicates modulation of the inflammatory response and suggests a role for monocytes in asthma pathogenesis.

## Background

Bronchial asthma, a chronic inflammatory disorder characterized by reversible airway obstruction and hyperresponsiveness [1], is one of the commonest chronic disorders in the world [2]. Its prevalence and symptoms vary in different geographical locations and it affects over two million people in Saudi Arabia. Although therapeutic options have improved, there are still unnecessary fatalities and there is a clear need to improve treatment strategies and define better biomarkers to identify patients at risk.

Allergic inflammation in asthma is characteristically associated with T helper (Th) cell and eosinophil infiltration in the bronchial mucosa [3]. Antigen-specific Th2 lymphocytes play a critical role in the generation of allergic inflammation through the release of cytokines, such as interleukin (IL)-4, IL-5, IL-9, and IL-13 [4, 5], which promote the activation and survival of eosinophils. However, monocytes are also likely to play a role due to increased production of monocyte-derived cytokines and mediators involved in oxidative stress, and in determining subsequent macrophage/dendritic cell and T helper cell phenotype and function [6, 7]. To date monocytes have received little attention in asthma pathogenesis although newly recruited monocyte-derived macrophages in the airway have been shown to be linked to eosinophilic airway inflammation [8]. Decrease in eosinophil recruitment in macrophage-depleted mice is thought to be due to reduction in macrophage chemokine production leading to altered recruitment of Th2 cells [8]. As a key immune cell in the bloodstream monocytes have been used to characterise severity of inflammation in other disorders including ischemic stroke and allergic rhinitis [7, 9, 10].

Human monocytes are heterogeneous and are classified into different subsets defined by the extent of their cell surface expression of CD14 and CD16, with associated differences in function and phenotype related to the intensity of expression of these markers. The major subset, termed classical monocytes, consists of CD14^high^CD16^negative^ (CD14^++^CD16^−^) monocytes, while the CD16 expressing monocytes are usually divided into a CD14^high^CD16^low^ (CD14^++^CD16^+^) intermediate subset and a CD14^low^CD16^high^ (CD14^+^CD16^++^) non-classical subset [11–13]. Differential expression of the chemokine receptors CCR2 and CX_3_CR1 is associated with these human monocyte subsets with the classical CD14^++^CD16^−^ subset predominantly expressing CCR2 and the non-classical CD14^+^CD16^++^ subset showing lower CCR2 expression and expressing significantly higher CX_3_CR1 [14–16].

These subsets have different functions with the CD14^++^CD16^−^ subset showing predominantly a phagocytic phenotype, CD14^++^CD16^+^ an inflammatory/antigen presenting phenotype and CD14^+^CD16^++^ a patrolling phenotype in blood vessels that also has ability to present antigen [17, 18]. The CD14^++^CD16^+^ and CD14^+^CD16^++^ subsets account for only 5–15% of all human monocytes, but their frequency increases significantly in certain inflammatory conditions [17, 19–21]. One study of patients with asthma in Poland showed a significant increase in the frequency of CD14^++^CD16^+^ monocytes in patients with severe asthma compared to healthy controls or patients with mild and moderate asthma [22] and it was suggested that this increase in the intermediate population might be a useful biomarker for asthma severity. However, whether this is universally applicable is unknown.

Our aims were firstly to test, in a different population, the hypothesis that increase in the percentage of intermediate monocytes is associated with severe asthma and secondly to determine whether expression of the chemokine receptors, CCR2 and CX_3_CR1, on monocyte subsets can act as indicators of asthma severity. We used flow cytometry to compare monocyte populations in Saudi Arabian adults with asthma and healthy age matched controls and analysed data to identify any associations between subset populations and asthma severity.

## Methods

### Participants

The participants were all from Saudi Arabia and included 35 healthy, non-smoking adult volunteers with no history of asthma or of any respiratory disease as controls (Table 1). None of the control subjects had suffered from any febrile illness during the last 3 months or were taking any medication. Seventy-six patients routinely attending asthma clinics while in a non-attack state were studied and presented with mild asthma (22 patients), moderate asthma (32 patients) and severe asthma (22 patients) (Tables 1 and 2).

**Table 1:** Demographic and clinical characteristics of subjects with asthma and healthy controls

	Healthy control	Asthma		
		Mild	Moderate	Severe
N	35	22	32	22
Mean, age ±SD	39 ± 12.86	39 ± 11.64	46 ± 16.1	52 ± 12.73
Gender, F|M	15|20	13|9	19|13	16|6
Asthma duration, years (mean)	–	18.33	16.03	21.48
Family history, n (%)	–	15 (68.2)	17 (53.1)	15 (68.2)
FEV%	–	93	77.38	69.25
History of smoking, n (%)	0	1 (4.5)	1 (3.1)	0
Current smoking, n (%)	0	3 (13.6)	3 (9.4)	2 (9.1)
SHS, n (%)		2 (9.1)	2 (6.25)	2 (9.1)
Treatment				
ICS/LABA combined inhaler				
Budesonide/formoterol (160 µg/4.5 µg)				
n (%)	0	0	23 (84.4)	15 (77.3)
n/day			2/day	2/day
Fluticasone/Salmeterol (125 µg/25 µg)				
n (%)	0	0	9 (28.1)	7 (31.8)
n/day			2/day	2/day
SABA				
Salbutamol (200 µg)				
n (%)	0	1 (4.5)	3 (9.4)	22 (100)
n/day		2/day	4/day	2/day
PRN		21 (95.4)	29 (90.6)	0
Tiotropium				
Spiriva (18 µg)				
n (%)	0	0	0	15 (68.2)
n/day				1/day
Montelukast				
Singulair (10 µg)				
n (%)	0	0	12 (37.5)	20 (90.9)
n/day, course			1 course	1/day
Anti-IgE				
Omalizumab (300 mg)				
n (%)	0	0	0	19 (86.4)
n/month				1/month

Data are presented in the form of numbers or percentage (%)

*FEV1* forced expiratory volume in 1 s; *SHS* second hand smoke; *ICS* inhaled corticosteroid; *LABA* long acting β_2_ agonist; *SABA* short acting β_2_ agonist; *PRN* as needed

**Table 2:** Co-morbidities and laboratory characteristics of asthma patients

Co-morbidities	Mild, n = 22 (%)	Moderate, n = 32 (%)	Severe, n = 22 (%)
Allergic rhinitis			
Yes	1 (4.5)	14 (43.75)	18 (81.81)
No	21 (95.45)	18 (56.25)	4 (18.19)
Hypertension			
Yes	0	7 (21.88)	11 (50)
No		25 (78.12)	11 (50)
Diabetes mellitus			
Yes	0	7 (21.88)	5 (22.73)
No		25 (78.12)	17 (77.27)
Obesity			
Yes	1 (4.5)	3 (9.38)	4 (18.19)
No	21 (95.45)	29 (90.62)	18 (81.81)
GERD			
Yes	0	11 (34.38)	7 (31.82)
No		21 (65.62)	15 (68.18)
Laboratory characteristics			
Total IgE^a^	–	32 (100) 0–100	8 (36.4) > 100
			9 (40.9) > 300
			5 (22.7) 500–700
% Eosinophil^b^	22 (100) 0–6	32 (100) 6.2–11	22 (100) 7.4–14
# Eosinophil^c^	22 (100) 0.2–0.8	32 (100) 9–1.23	22 (100) 1.1–1.5
% Neutrophil^d^	22 (100) 40–75	28 (87.5) 40–75	20 (90.9) 40–75
		2 (6.25) > 75	1 (4.55) > 75
		2 (6.25) < 40	1 (4.55) < 40
# Neutrophil^e^	22 (100) 2–7.5	28 (87.5) 2–7.5	21 (95.45) 2–7.5
		2 (6.25) > 7.5	1 (4.55) < 2
		2 (6.25) < 2	
% Lymphocyte^f^	22 (100) 20–45	26 (81.25) 20–45	18 (81.81) 20–45
		1 (3.13) > 45	1 (4.55) > 45
		5 (15.62) < 20	3 (13.64) < 20
# Lymphocyte^g^	22 (100) 1–5	30 (93.75) 1–5	20 (90.9) 1–5
		2 (6.25) < 1	1 (4.55) > 5
			1 (4.55) < 1
% Monocyte^h^	22 (100) 3–9	25 (78.13) 3–9	17 (77.27) 3–9
		7 (21.87) > 9	5 (22.73) > 9
# Monocyte^i^	22 (100) 0.2–0.8	28 (87.5) 0.2–0.8	21 (95.45) 0.2–0.8
		4 (12.5) > 0.8	1 (4.55) < 0.8

Data are presented in the form of numbers or percentage (%)

*n* number of patients; Percentage of number of patients is shown in parentheses. *GERD* gastroesophageal reflux disease

“–” was not measured for mild asthma patients

^a^Total IgE normal range, 0–100 Ku/l

^b^Eosinophil percentage of leukocyte count normal range, 0–6%

^c^Eosinophil count normal range, 0.2–0.8 × 10^9^/l

^d^Neutrophil percentage normal range, 40–75

^e^Neutrophil count normal range, 2–7.5 × 10^9^/l

^f^Lymphocyte percentage normal range, 20–45

^g^Lymphocyte count normal range, 1–5 × 10^9^/l

^h^Monocyte percentage normal range, (3–9)

^i^Monocyte count normal range 0.2–0.8 × 10^9^/l

Severity of asthma was classified according to the Saudi Initiative for Asthma (SINA) guidelines based on Global Initiative for Asthma (GINA) criteria [23, 24]. Assessment of asthma severity was based on the treatment steps required to control symptoms and exacerbations.

*Mild asthma* controlled asthma at step 1 or 2 that needs reliever treatment, monotherapy of low-dose inhaled corticosteroids (ICS), or leukotriene receptor antagonist (LTRA).

*Moderate asthma* controlled asthma where the patients are on combination of ICS/long-acting beta 2 agonist (LABA) or other alternative options at step 3.

*Severe asthma* severe uncontrolled asthma at presentation (step 4 or 5) where patients require treatment with combination of high-dose ICS/LABA with or without add-on treatment.

### Ethical standards

All participants with asthma were selected from the respiratory outpatient clinic at King Khalid University Hospital. The study protocol was approved by the Institutional Review Board of King Khalid University Hospital, Ethics Committee, and signed informed consent was obtained from all participants.

### Flow cytometry

#### Sample collection and preparation

Five ml of venous blood was withdrawn from the cubital vein into EDTA-anticoagulant, transferred to a 50 ml centrifuge tube and centrifuged for 4 min at 431×*g*. The supernatant was discarded and 10 ml of red blood cell lysing solution (Sigma Aldrich R7757) was added and incubated for 20 min at room temperature (RT) followed by centrifugation for 4 min at 431×*g*. Re-suspended cell pellets were washed twice with 15 ml of FACS buffer (phosphate buffered saline (PBS) and 2% fetal bovine serum FBS) for 4 min at 431×*g* and re-suspended in 1 ml of FACS buffer.

#### Compensation, optimization and controls

Cytometer setup tracking (CST) research beads were used as a quality control for the instrument to improve the automated cytometer setup and performance. Compensation beads were used to ensure the integrity of the fluorochromes being used in the experiment before data acquisition. To optimize the fluorescence for the multi-color flow cytometric analysis, fluorescence compensation was run first for each fluorochrome being analysed.

In order to achieve consistent and reproducible results, and to minimize bleeding between the different dyes, optimization experiments were performed for surface markers (Alexa-700, BV510, BV421, Alexa-647, PE, and DAPI) prior to sample acquisition.

FMOs (fluorescence minus one) for each monoclonal antibody were prepared and run with the first batch of samples each week. An unstained cell suspension for each sample was included with each run. Appropriate isotype controls were also included (Table 3) to set up appropriate gating.

**Table 3:** Fluorescently labelled antibodies for monocyte cell surface markers (all mouse anti-human antibodies except rat anti-human)

Epitope	Conjugate	Manufacturer	Clone	Isotype	Test volume (µl)
CD45	AF700	Becton–Dickinson	HI30	IgG1, _k_	5
CD14	BV510	Becton–Dickinson	MφP9	IgG2b, _k_	5
CD16	BV421	Becton–Dickinson	3G8	IgG1, _k_	3
CCR2	AF647	Becton–Dickinson	48607	IgG2b	5
CX3CR1 (rat anti-human)	PE	MBL	2A9-1	IgG2b, _k_	10

#### Cell surface staining

Aliquots of each cell suspension sample (100 µl) were incubated (30 min) at 4 °C in the dark with anti-CD45, anti-CD14, anti-CD16, anti-CD192 (CCR2) and anti-CX_3_CR1 fluorescently conjugated monoclonal antibodies (Table 3). Following incubation, cells were washed with 1 ml FACS buffer followed by centrifugation at 431×*g* for 4 min. The cell pellets were re-suspended in 500 µl of FACS buffer and 3×10^5^ cells were acquired using LSRII flow cytometer (BD Biosciences). Data analysis was performed using FACS Diva software (BD Biosciences).

#### Gating strategy

**Fig. 1:**
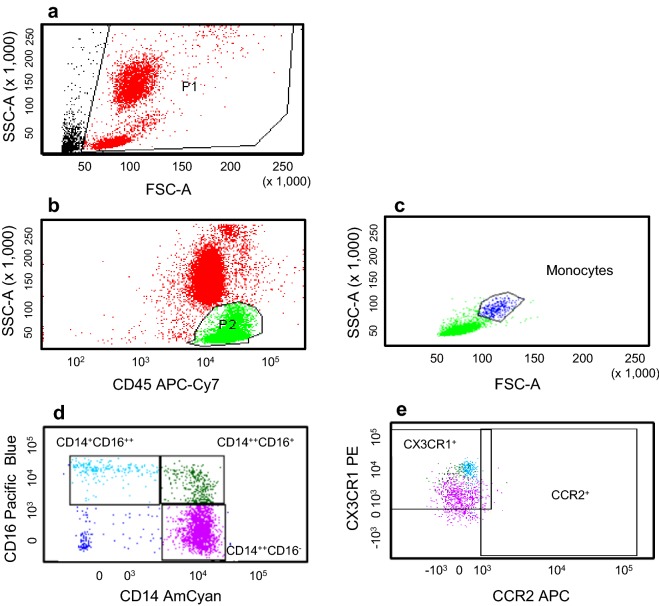
Gating strategy for defining monocyte subsets. **a** A side scatter (SSC) versus forward side scatter (FSC) dot plot was used to identify cells from debris (P1); **b** a SSC/CD45 dot plot from live gate (P2) was used to gate on CD45^+^ cells and exclude large granulocytes; **c** a FSC/SSC dot plot from the CD45^+^ expressing cells was used to tightly gate on the monocyte population (positioned just above the lymphocytes); **d** a CD14/CD16 dot plot from CD14^+^ monocytes was arbitrarily set based on their fluorescent intensity and on FMO and isotype controls to reveal the three monocyte subsets (CD14^++^CD16^−^, CD14^++^CD16^+,^ and CD14^+^CD16^++^); **e** the expression of CCR2 and CX_3_CR1 by the three monocyte subsets was set based on FMO and isotype control

First, forward scatter (FSC) and side scatter (SSC) dot plots were used to identify cells from debris. An SSC/CD45 dot plot from a live gate was used to gate on CD45^+^cells and exclude large granulocytes. An FSC/SSC dot plot from the CD45^+^ expressing cells was used to tightly gate on the monocyte population as previously described by our group [25]. A CD14/CD16 dot plot was expressed from the gated monocytes to reveal the three monocyte subsets (CD14^++^CD16^−^, CD14^++^CD16^+,^ and CD14^+^CD16^++^) based on their fluorescent intensity based on FMO and isotype controls. A CCR2/CX_3_CR1 dot plot was obtained for each monocyte subset (Fig. 1).

### Statistical analysis

Analysis was performed through Graph Pad PRISM version 6 (Graph Pad software, La Jolla, CA, USA). Significance was calculated using a Holm–Sidak unpaired comparison test. Results were considered statistically significant if the *p* value was < 0.05. Mean percentages are given in the text ± standard error. Correlations were analysed using nonparametric Spearman’s rank correlation coefficient.

## Results

### Expression of CD16 is altered on monocyte subsets in patients with asthma

**Fig. 2:**
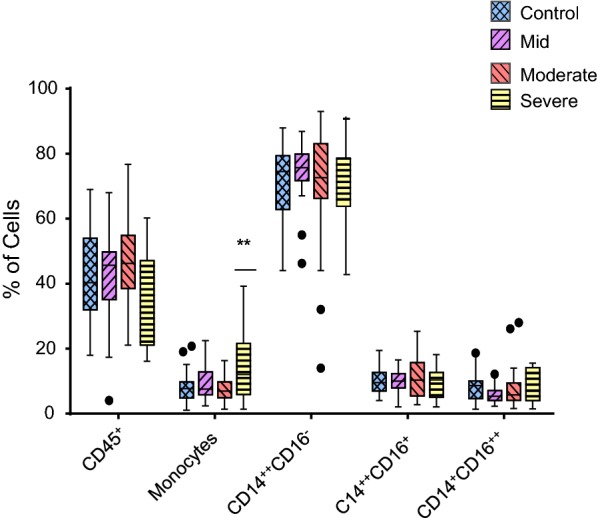
Comparative analysis of the percentage of CD45^+^ cells, total monocyte population and monocyte subsets in mild asthmatic patients (n = 22), moderate asthmatic patients (n = 32) and severe asthmatic patients (n = 22) compared to healthy control patients (n = 35). Each monocyte subset is shown as box groups where the left box indicates healthy subjects and the next boxes indicate mild, moderate and severe asthmatic patients respectively. The box ranges from the 25th to 75th percentile, where the top end of the whiskers indicates the largest value less than the sum of 75th percentile plus 1.5×IQR (interquartile range) and any values greater than this are considered outliers (represented by dots) and the bottom end of the whiskers indicates the lowest value greater than the 25th percentile minus 1.5×IQR and any values less than this are considered outliers. The middle line represents the median. Significance was calculated using Holm–Sidak unpaired multiple comparison test and is indicated with **(*p *< 0.01)

The percentage of blood monocytes was significantly increased in patients with severe asthma (15 ± 2; p = 0.002) compared to healthy individuals (8%), while no significant change was observed in the patients with mild (9 ± 1) and moderate (7 ± 1) asthma (Fig. 2). The proportion of the three individual monocyte subsets showed no significant changes in patients with mild, moderate and severe asthma compared with healthy individuals (Fig. 2).

**Fig. 3:**
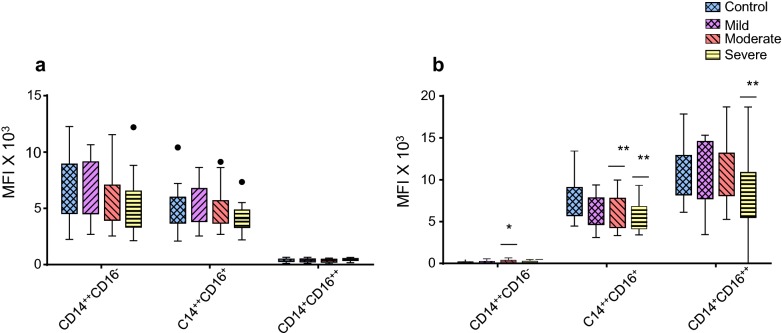
Comparative analysis of CD14 expression (**a**) and CD16 expression (**b**) on monocyte subsets from mild asthmatic patients (n = 22), moderate asthmatic patients (n = 32) and severe asthmatic patients (n = 22) compared to healthy control (n = 35). Each monocyte subset is shown as box groups (as defined in Fig. 2) where the left box indicates healthy subjects and the next boxes indicate mild, moderate and severe asthmatic patients respectively. Gating was performed as in Fig. 1 and significance, compared to healthy controls, was calculated using Holm–Sidak unpaired multiple comparison test and is indicated with *(*p *< 0.05), **(*p *< 0.01)

Our data showed that the expression level (MFI) of CD14 on the monocyte subsets was not significantly different in patients with mild, moderate and severe asthma compared to healthy controls (Fig. 3a). However, the expression level of CD16 on CD14^++^CD16^+^ intermediate subset monocytes was significantly lower in patients with moderate (p = 0.006) and severe (p = 0.004) asthma and there was also a significant decrease (p = 0.005) in CD16 expression on the non-classical CD14^+^CD16^++^ monocyte subset in patients with severe asthma compared to healthy controls (Fig. 3b). Collectively, these data demonstrated that while the percentage of the monocyte subsets was not significantly changed, there was differential expression of CD16 on monocyte subsets associated with asthma severity.

### CCR2 and CX_3_CR1 chemokine receptor expression is altered on monocytes from patients with asthma

**Fig. 4:**
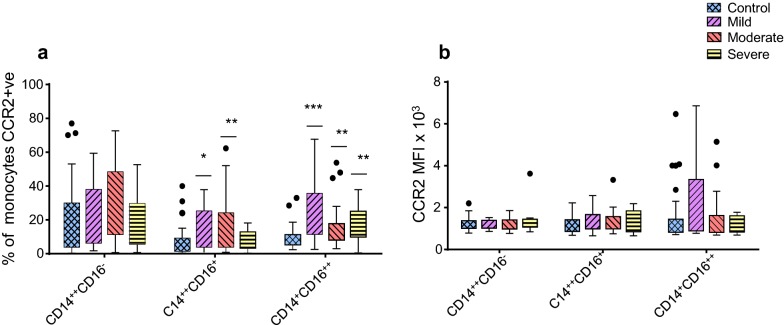
Comparative analysis of the percentage of monocytes that are CCR2 positive (**a**) or CCR2 expression (MFI; **b**) on monocyte subsets from mild asthmatic patients (n = 22), moderate asthmatic patients (n = 32) and severe asthmatic patients (n = 22) compared to healthy control patients (n = 35). Each monocyte subset is shown as box groups (as defined in Fig. 2^2^ where the left box indicates healthy subjects and the next boxes indicate mild, moderate and severe asthmatic patients respectively. Gating was performed as in Fig. 1 and significance, compared to healthy controls, was calculated using Holm–Sidak unpaired multiple comparison test and is indicated with **(*p *< 0.01), ***(*p *< 0.001)

Expression of CCR2, is associated with phagocytic monocytes [14–17]. In the non-classical CD14^+^CD16^++^ subset, the percentage of monocytes expressing CCR2 was significantly higher in the patients with mild (24 ± 3; p < 0.0001), moderate (16 ± 2.5; p = 0.009) and severe (18 ± 2.7; p = 0.002) asthma compared to healthy (9%) controls (Fig. 4a). The percentage of monocytes expressing CCR2 in the intermediate CD14^++^CD16^+^ subset was significantly higher only in patients with mild (17 ± 4; p = 0.018) and moderate (17 ± 3; p = 0.003) asthma, not severe asthma, compared to healthy (7%) controls (Fig. 4a). The percentage of monocytes expressing CCR2 in the classical CD14^++^CD16^−^ subset was not changed when patients with mild, moderate or severe asthma were compared with healthy controls (Fig. 4a). The MFI for CCR2 on all monocyte subsets showed no significant difference in patients with mild, moderate or severe asthma compared to healthy controls (Fig. 4b).

**Fig. 5:**
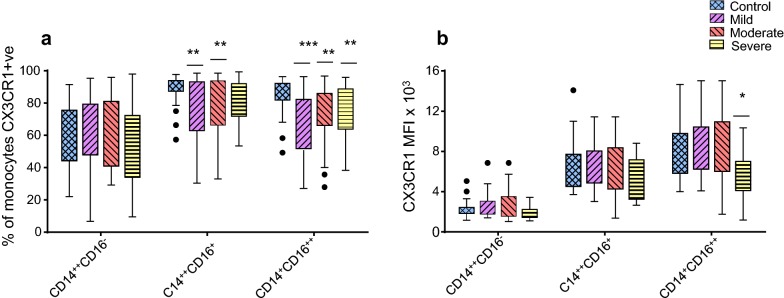
Comparative analysis of the percentage of monocytes that are CX_3_CR1 positive (**a)** or CX_3_CR1 expression (MFI; **b**) on monocyte subsets from mild asthmatic patients (n = 22), moderate asthmatic patients (n = 32) and severe asthmatic patients (n = 22) compared to healthy control patients (n = 35). Each monocyte subset is shown as box groups (as defined in Fig. 2) where the left box indicates healthy subjects and the next boxes indicate mild, moderate and severe asthmatic patients respectively. Gating was performed as in Fig. 1 and significance, compared to healthy controls, was calculated using Holm–Sidak unpaired multiple comparison test and is indicated with *(*p *< 0.05), **(*p *< 0.01), ***(*p *< 0.001)

**Fig. 6:**
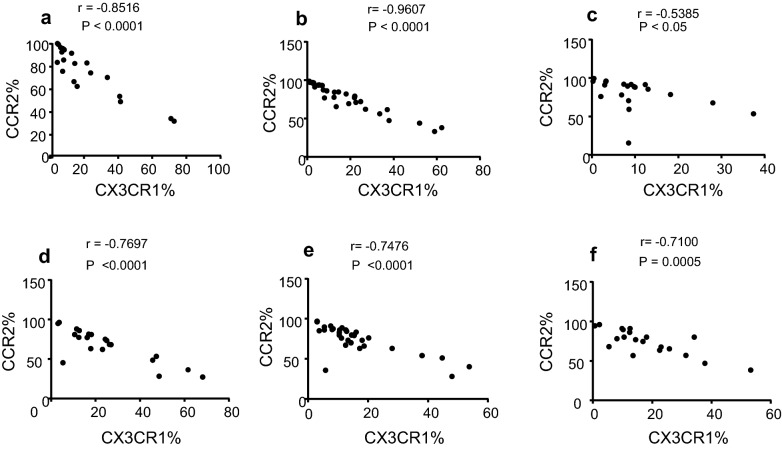
Correlations shown are between the percentage of monocytes that are CCR2 or CX3CR1 positive in the CD14^++^CD16^+^ monocyte subset from mild (**a**) moderate (**b**) and severe (**c**) asthmatic patients and in the CD14^+^CD16^++^ monocyte subset from mild (**d**) moderate (**e**) and severe (**f**) asthmatic patients using nonparametric Spearman rank correlation R_s_

Expression of CX_3_CR1 is associated with patrolling monocytes [14–17]. In the non-classical CD14^+^CD16^++^ subset, the percentage of cells expressing CX_3_CR1 was significantly decreased in patients with mild (67 ± 4; p < 0.0001, moderate (74 ± 3.5; p = 0.003) or severe (74 ± 3.6; p = 0.003) asthma compared to healthy controls (85%) (Fig. 5a). A significant decrease was observed in the percentage of cells expressing CX_3_CR1 in the intermediate CD14^++^CD16^+^ subset in patients with mild (75 ± 4; p = 0.002) and moderate (78 ± 3; p = 0.004) asthma compared to healthy (88%) controls (Fig. 5a). The percentage of monocytes expressing CX_3_CR1 in the classical CD14^++^CD16^−^ subset was not changed when patients with mild, moderate or severe asthma were compared with healthy controls (Fig. 5a). CX_3_CR1 MFI was also significantly decreased (p = 0.0146) on the non-classical CD14^+^CD16^++^ subset in patients with severe asthma compared to healthy control subjects (Fig. 5b). Together, all patients with asthma demonstrated a significant (*p *< 0.05) inverse relationship between CCR2 (increased compared to controls) and CX_3_CR1 (decreased compared to controls) percentage expression in the intermediate CD14^++^CD16^+^ subset (Fig. 6a–c) although this relationship was weaker in the severely asthmatic patients (Fig. 6c). For the non-classical CD14^+^CD16^++^ subset all the patients strongly showed this inverse relationship with Spearman’s rank correlation coefficient analysis showing this to be highly significant (*p *≤ 0.0005, Fig. 6d–f).

## Discussion

In the presented work, we have studied monocytes from patients with asthma (all from Saudi Arabia) in terms of their CD14/CD16 defined subsets and expression of the chemokine receptors CCR2 and CX_3_CR1 to determine whether there are monocyte characteristics that can be linked to asthma or to the disease severity.

### Proportions of monocyte subsets did not change with asthma severity

Our study showed a significant increase in the percentage of the CD45^+^ population of blood cells that were monocytes (CD45^+^/CD14^+^) in patients with severe asthma compared to healthy controls. The observed increase in this percentage of monocytes in patients with severe asthma may reflect their higher susceptibility to an allergenic or stress response and subsequent inflammatory reaction and release of monocytes from bone marrow or spleen [26, 27].

Despite the increase in the percentage of the CD45^+^ population that were monocytes, the proportions of classical, intermediate and non-classical monocyte subsets, as defined by CD14/CD16 expression, showed no significant difference in patients compared to healthy controls. While most asthma studies have focused on assessing the total monocyte numbers, one study in particular, by Moniuszko et al. [22], demonstrated a significant increase in the intermediate CD14^++^CD16^+^ monocyte percentage in patients with severe asthma compared to healthy controls or to patients with mild and moderate asthma [22]. Our study, however, provides no evidence that the proportion of this intermediate subset is increased with severe asthma. This may be a result of gating variation between the two studies; for example, our study gated on CD45^+^ cells initially. Perhaps more importantly, one of the main dissimilarities is that the study by Moniuszko et al. [22] involved patients from Poland whereas the patients in our study are from Saudi Arabia, where asthma may be triggered by different environmental factors. This suggests that differences may also reflect genetic or environmental factors influencing the distinct populations.

### Decreased expression of CD16 on monocyte subsets in asthma

There was a significant decrease in CD16 MFI expression in patients with moderate and severe asthma in the intermediate (CD14^++^CD16^+^) monocyte subset and also in the non-classical (CD14^+^CD16^++^) subset in patients with severe asthma compared to healthy controls. Changes in CD16 MFI have not been investigated in previous asthma studies although reduction in CD16 MFI on monocytes has been reported in other patients, for example, in patients with coronary artery disease [28].

CD16^+^ monocytes are often expanded in inflammatory conditions such as sepsis and atherosclerosis [17, 21, 29]. It has been shown with human monocyte subsets that monocytes are released from the bone marrow in an inflammatory response as classical monocytes and differentiate sequentially in the circulation into intermediate and then non-classical monocytes [30]. This suggests that the decreased CD16^+^ expression we show, on intermediate and non-classical monocytes, associated with increased asthma severity may be due to the fact that monocytes are recently released from the bone marrow and have not yet acquired full CD16 expression.

CD16 on human monocytes is the Fc receptor, FcγRIIIa (CD16a) which is a low affinity IgG receptor [31] unlikely to promote an inflammatory response in asthma. However, CD16^+^ monocyte subsets have distinct roles in the inflammatory response, with the non-classical subset responsible for a patrolling, ‘house-keeping’ role, able to attach and crawl on endothelium and distinguish virally infected and damaged cells [32].

### Chemokine receptor expression on monocyte subsets changes in patients with asthma

In addition to CD14 and CD16 surface expression, monocyte subpopulations can be further characterised based on the expression of chemokine receptors, with the phagocytic classical monocyte subset expressing high levels of CCR2 and the non-classical patrolling subset expressing low CCR2 and high CX_3_CR1 [14, 33, 34].

In this study, analysis of the percentage of cells expressing CCR2 and CX_3_CR1 in the intermediate and non-classical monocyte subsets shows a strong inverse correlation, with CCR2 increased and CX_3_CR1 decreased, in patients with asthma compared to healthy controls. This is consistent with our finding that monocyte subsets have decreased CD16^+^ associated with increased asthma severity and the suggestion that monocytes newly released from the bone marrow have not fully reduced CCR2 and acquired CX_3_CR1 in addition to CD16.

CCR2 and its ligands are known to be crucial for the recruitment of inflammatory monocytes, enhancing monocyte adhesion to endothelium leading to their exit into the site of inflammation [35–37]. In response to infection CCR2-positive monocytes can be recruited in large numbers and in the lung in particular this can cause widespread damage as they appear to be less likely than in the skin for example to convert to M2 macrophages associated with wound healing and repair [38].

In the non-classical (CD14^+^CD16^++^) monocyte subset, which patrols the endothelium, clearing debris and scanning for an inflammatory stimulus, CCR2 is down-regulated and CX_3_CR1 is highly expressed with CX_3_CR1 linked with patrolling ability and survival [18–20]. A general decrease in the frequency or expression of CX_3_CR1 on human monocytes has been shown in other conditions, for example, in patients with atopic dermatitis [39]. Thus these changes in CCR2 and CX_3_CR1 expression in intermediate and non-classical monocyte subsets indicate a more phagocytic phenotype and increased likelihood of endothelial damage adversely affecting asthma progression.

One unavoidable limitation is that the patients included in this study were receiving treatment to reduce inflammation (Table 1) and it is possible that the treatment will have influenced monocyte characteristics. However, despite this, significant changes in CCR2 and CX_3_CR1 on the intermediate and non-classical monocyte subsets were still evident however, whether they are a cause or consequence of disease remains to be established.

## Conclusions

This differential expression of CD16, CX_3_CR1 and CCR2 on the monocyte subsets in peripheral blood indicates that monocytes may modulate the inflammatory response in asthma. The increase in CCR2 expression and decrease in CX_3_CR1 on CD16^+^ monocytes has not, to our knowledge, been described before in asthma and this association suggests a role for monocytes in asthma pathogenesis.

## Data Availability

The datasets analysed during the current study are available from Dr. Reem AlRashoudi on reasonable request.

## Abbreviations

ICS: inhaled corticosteroids
LTRA: leukotriene receptor antagonist
LABA: long-acting beta 2 agonist
FMOs: fluorescence minus ones
DCs: dendritic cells
SINA: Saudi Initiative for Asthma
GINA: Global Initiative for Asthma
Th: T helper
IL: interleukin
MFI: mean fluorescence intensity
